# Isolated Splenic Tuberculosis in an Immunocompetent Patient: A Rare Case

**DOI:** 10.7759/cureus.13463

**Published:** 2021-02-21

**Authors:** Mohamed Chablou, Rachid Jabi, Ahmed Id M'barek, Noura Seghrouchni, Mohammed Bouziane

**Affiliations:** 1 General Surgery, Mohammed VI University Hospital/Faculty of Medicine and Pharmacy of Oujda, Mohammed First University of Oujda, Oujda, MAR; 2 Pulmonary Medicine, Mohammed VI University Hospital/Faculty of Medicine and Pharmacy of Oujda, Mohammed First University of Oujda, Oujda, MAR; 3 Pathology, Mohammed VI University Hospital/Faculty of Medicine and Pharmacy of Oujda, Mohammed First University of Oujda, Oujda, MAR

**Keywords:** splenic tuberculosis, tuberculosis, miliary, disseminated tuberculosis

## Abstract

Tuberculosis (TB) is one of the top 10 causes of death worldwide and the leading cause of death from a single infectious agent. Despite early diagnosis and improvements in medical science, the incidence of the disease is still a major public health problem in developing countries. Splenic tuberculosis is quite rare and occurs mostly as a part of miliary tuberculosis in individuals with immunosuppression. Isolated splenic tuberculosis is extremely rare in immunocompetent patients. We report a case of an immunocompetent man with isolated splenic tuberculosis.

## Introduction

Tuberculosis is defined as a microbial, contagious disease caused by infection with Mycobacterium tuberculosis. It remains a real public health problem in developing countries [1]. Extrapulmonary tuberculosis accounts for almost 16% of all cases [2]. Isolated splenic tuberculosis is extremely rare, particularly in immunocompetent persons. It is normally seen as part of miliary tuberculosis. The incidence of splenic tuberculosis may be variable depending on the prevalence of the disease in a particular geographical area [3]. The present series reported 4%-12% of splenic tuberculosis in patients subjected to a diagnostic splenectomy[3]. In this case report, we report a rare case of 62-year-old men with solitary splenic tuberculosis.

## Case presentation

A 62-year-old man presented to our outpatient service with left hypochondriac pain radiating to the shoulder associated with low-grade fever, anorexia, and weight loss for five weeks’ duration. There was no history of cough, breathlessness, chest pain, or hemoptysis. His medical history did not include any tuberculosis.

On clinical examination, his body temperature was 38.3 °C. Abdominal palpation revealed mildly enlarged and tender spleen without any other significant findings.

His hemogram revealed normocytic normochromic anemia (hemoglobin: 9 g/dl); leukocyte and platelet counts were normal. Liver and renal function tests were within normal limits. C-reactive protein (CRP) was elevated (130 mg/l). Hepatitis B, hepatitis C, and human immunodeficiency virus (HIV) markers were negative. Chest X-ray was unremarkable.

**Figure 1:**
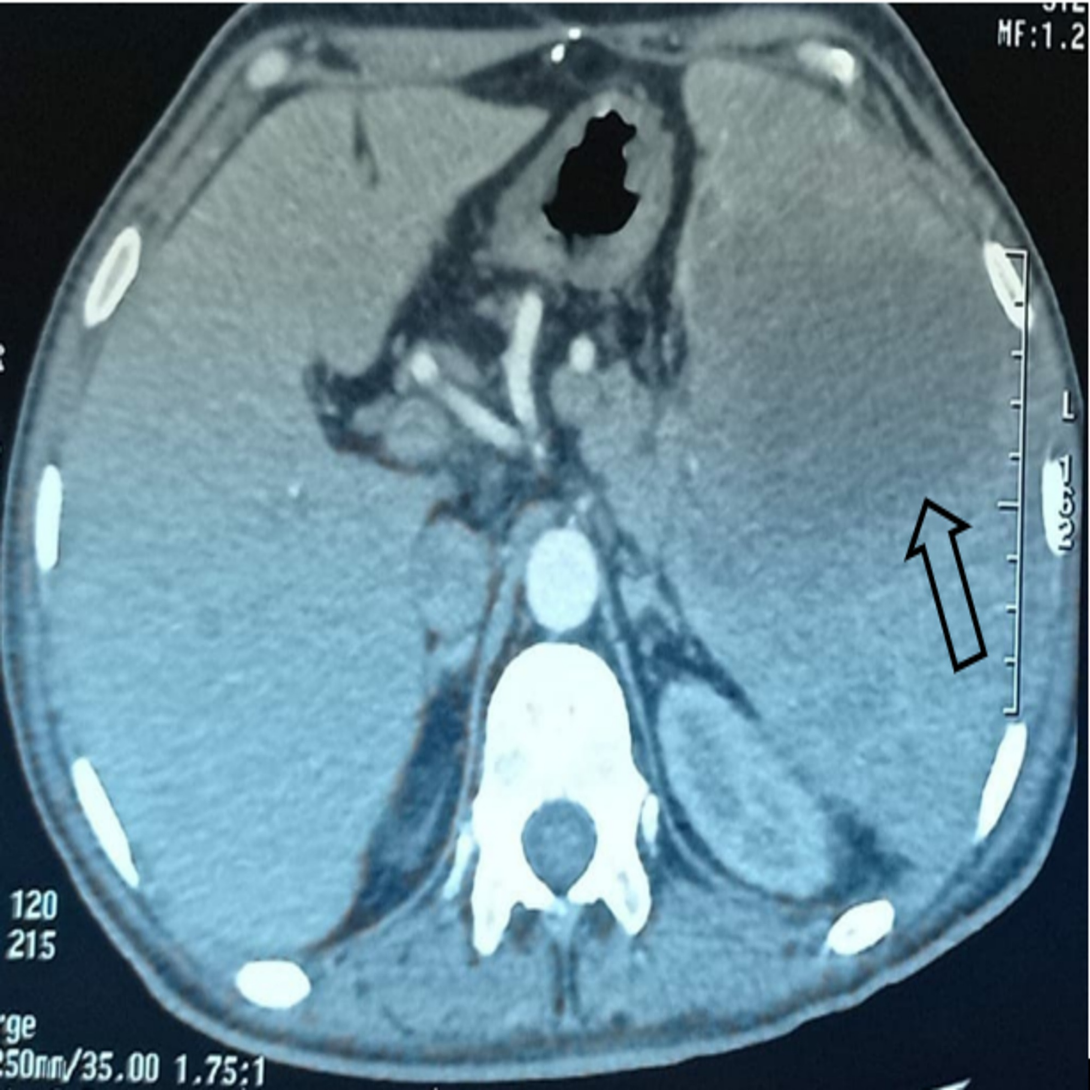
Computed tomography scan showing hypodense areas within the spleen (black arrow)

Abdominal ultrasound showed splenomegaly with the presence of multiple hypo-echoic lesions. Abdominal computed tomography (CT) scan showed a heterogeneous enlarged spleen measuring 168 mm in size, with multiple hypodense areas, some of which contain centrally irregular necrosis, the largest at the mid-splenic level, measuring 65x57 mm, associated with infiltration of perisplenic fat (Figure 1).

**Figure 2:**
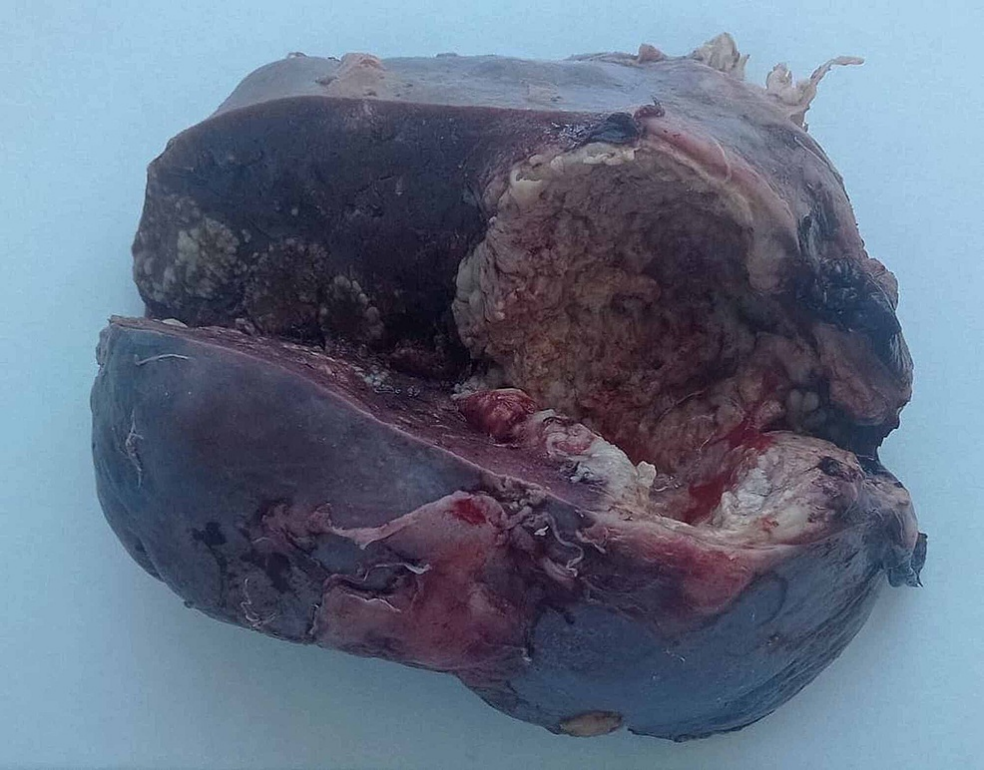
Image of the surgical specimen: large spleen with intra-splenic abscess

Fine-needle aspiration was not performed, as it was technically unfeasible. Then, the patient underwent open splenectomy for diagnostic and therapeutic purposes (Figure 2).

A histologic report showed splenic parenchyma reshaped by a granulomatous inflammatory infiltrate with large confluent epithelioid granulomas, associated with the presence of giant cells with foci of caseation necrosis. Ziehl-neelsen stain did reveal *Mycobacterium tuberculosis* and was compatible with splenic tuberculosis.

Therefore, a final diagnosis of splenic tuberculosis was made, and the patient was started on quadruple anti-tuberculosis therapy four drugs for two months: isoniazid (5 mg/kg), rifampicin (10 mg/kg), pyrazinamide (25 mg/kg), and ethambutol (20 mg/kg), and then two drugs for the next four months: isoniazid and rifampicin. The patient remained asymptomatic after a six-month follow-up with an improvement of his general condition.

## Discussion

Abdominal tuberculosis is an infection caused by *Mycobacterium tuberculosis* that affects the gastrointestinal and urinary tract, as well as the peritoneum, lymph nodes, or solid organs (liver, spleen, and pancreas) [4]. The World Health Organization estimates that extrapulmonary tuberculosis accounts for about 16% of all tuberculosis cases in 2019 [2].

Isolated splenic tuberculosis is very rare in immunocompetent subjects, and more frequent in immunocompromised patients, principally in HIV-infected patients [5].

Spleen is the third most common organ (lung 100%, liver 82%, spleen 75%, lymph nodes 55%, and bone marrow 41%) involved in miliary tuberculosis [6-7].

Our patient was immunocompetent. He had neither a history of TB nor showed evidence of TB in any other organ and no immunosuppressive condition. The diagnosis of splenic tuberculosis is difficult because of non-specific clinical manifestations. In our case, the chief complaints and symptoms were abdominal pain associated with low-grade fever and weight loss. In other case reports of splenic tuberculosis, the principal symptom was abdominal discomfort, without fever or weight loss [8-9].

Laboratory data, which are often disturbed, do not provide certain elements in favor of the diagnosis. On the hemogram, signs of hypersplenism are present in 35% [10], associated anemia, leukopenia, even thrombocytopenia, which is not the case of our patient, apart from normocytic normochromic anemia at 9 g/dl, these disturbances of the hemogram can be observed in other hemopoietic pathologies such as lymphoma and leukemia. Thus, high CRP can be seen in pyogenic abscesses and hydatid cysts.

Ultrasound allows confirmation of clinically uncertain splenomegaly, detection, and characterization of focal lesions, objectification of other associated pathologies, and guidance of fine-needle aspiration [11]. Three ultrasound imaging aspects have been described: the micronodular or miliary form is rarely diagnosed at an early stage [12], the macronodular form that corresponds either to tubers in focus or cold abscesses, and the pseudotumoral form. In our patient, abdominal ultrasound showed splenomegaly with the presence of multiple hypo-echoic lesions.

The abdominal CT scan allows a precise topographical assessment of the splenic lesions and associated regional damage; in our case, the splenic damage was isolated. Thus, the sensitivity of the scanner in splenic abscesses is around 90% to 100% [13]. However, splenic abscesses of tubercular origin can pose a diagnostic problem with pyogenic abscesses, primary tumors, and hydatid cysts [14-15].

The non-specificity of the clinical, biological, and radiological signs makes histological examination essential for the diagnosis of an isolated splenic lesion especially by fine-needle aspirate, splenic biopsy, or splenectomy. The fine needle aspiration cytology is a valuable tool, with a sensitivity of 88% and specificity of up to 100% [16]. In our case, fine-needle aspiration was not performed, as it was technically unfeasible.

A final diagnosis of splenic tuberculosis was made upon histopathological findings, and quadruple antitubercular therapy (isoniazid, rifampicin, pyrazinamide, and ethambutol) was started for two months followed by four months of isoniazid and rifampicin with an improvement in the general condition of the patient [17].

## Conclusions

Isolated splenic tuberculosis remains rare, even in countries where tuberculosis is endemic such as Morocco. It poses a problem of differential diagnosis, linked to clinical polymorphism and the lack of biological and radiological specificity. Directed biopsy in these cases is of great diagnostic and sometimes therapeutic interest, making it possible to avoid unnecessary splenectomies. When the diagnosis of splenic tuberculosis is made preoperatively, splenectomy could be considered for therapeutic purposes to eradicate a septic source resistant to medical treatment or in the case of complications such as splenic rupture. It could be considered for diagnostic purposes when percutaneous procedures are contraindicated (hemostasis disorder), inconclusive, or technically unfeasible as seen in our case.
